# Impaired lower limb muscle mass, quality and function in end stage liver disease: A cross‐sectional study

**DOI:** 10.1113/EP091157

**Published:** 2023-05-11

**Authors:** Jonathan I. Quinlan, Amritpal Dhaliwal, Felicity R. Williams, Sophie L. Allen, Surabhi Choudhary, Alex Rowlands, Leigh Breen, Gareth G. Lavery, Janet M. Lord, Ahmed M. Elsharkawy, Matthew J. Armstrong, Carolyn A. Greig

**Affiliations:** ^1^ NIHR Birmingham Biomedical Research Centre University Hospitals Birmingham NHS Foundation Trust and University of Birmingham Birmingham UK; ^2^ School of Sport, Exercise and Rehabilitation Sciences University of Birmingham Birmingham UK; ^3^ Institute of Inflammation and Ageing University of Birmingham Birmingham UK; ^4^ Therapies Department University Hospitals Birmingham Birmingham UK; ^5^ Department of Imaging University Hospitals Birmingham Birmingham UK; ^6^ NIHR Leicester Biomedical Research Centre Leicester UK; ^7^ Diabetes Research Centre University of Leicester, Leicester General Hospital Leicester UK; ^8^ MRC‐Versus Arthritis Centre for Musculoskeletal Ageing Research University of Birmingham Birmingham UK; ^9^ Department of Biosciences Nottingham Trent University Nottingham UK; ^10^ Liver Unit Queen Elizabeth Hospital Birmingham Birmingham UK

**Keywords:** cirrhosis, frailty, liver, myosteatosis, sarcopenia

## Abstract

Sarcopenia is associated with reduced quality of life and increased mortality in patients with end stage liver disease (ESLD). Historically, sarcopenia identification in ESLD utilised L3 skeletal muscle index (SMI). There are few data on muscle quality and function within lower limb muscle groups with high functional relevance. The aim of this prospective case–control study was to evaluate the quadriceps muscle in patients with ESLD. Muscle mass and quality were evaluated using MRI (quadriceps anatomical cross sectional area (ACSA), quadriceps volume index, L3 SMI, quadriceps intermuscular adipose tissue (IMAT)), mid‐arm muscle circumference (MAMC) and ultrasonography (vastus lateralis (VL) thickness and quadriceps ACSA). Muscle strength/function was assessed by handgrip strength, peak quadriceps isokinetic torque and chair rise time. Thirty‐nine patients with ESLD (55 years, 61% male, 48% alcoholic related liver disease (ArLD), 71% Child–Pugh B/C) and 18 age/sex‐matched healthy control participants (HC) were studied. Quadriceps mass was significantly reduced in ESLD versus HC (−17%), but L3 SMI and MAMC were unchanged. Quadriceps IMAT percentage was increased in ESLD (+103%). Handgrip strength (−15%), peak isokinetic torque (−29%), and chair rise time (+56%) were impaired in ESLD. Ultrasound measures of VL thickness (*r* = 0.56, *r* = 0.57, *r* = 0.42) and quadriceps ACSA (*r* = 0.98, *r* = 0.86, *r* = 0.67) correlated to MRI quadriceps ACSA, quadriceps volume and L3 SMI, respectively. Quadriceps muscle mass, quality, and function were impaired in patients with ESLD, whereas conventional assessments of muscle (L3 SMI and MAMC) highlighted no differences between ESLD and HC. Full evaluation of lower limb muscle health is essential in ESLD in order to accurately assess sarcopenia and target future interventions.

## Abstract (highlights)

- **What is the central question of this study?**
  To what extent does musculoskeletal impairment occur (i.e., muscle mass, quality and function) in patients with end stage liver disease (ESLD) by comparison to a healthy age/sex‐matched control group?
- **What is the main finding and its importance?**
  Muscle mass, quality and function are impaired in patients with ESLD (compared to age/sex matched controls). Importantly, greater impairments were seen in lower limb compared to arm and trunk muscle groups. These findings may suggest that there should be greater consideration of muscle health in functionally relevant lower limb muscle groups.

## 1 INTRODUCTION

Sarcopenia affects approximately 22–70% of patients with end stage liver disease (ESLD) (Kim et al., 2017) and it can have a negative impact on quality of life and risk of mortality and adversely affects the outcome of liver transplantation (Carey et al., 2017). There has been much debate around the definition of sarcopenia and similar related terms, such as malnutrition and frailty, in patients with ESLD (Lai et al., 2021; Williams et al., 2021). The American Association for the Study of Liver Disease (AASLD) has defined sarcopenia as the phenotypic manifestation of loss of muscle mass alone (Lai et al., 2021). As such, the assessment of sarcopenia in patients with ESLD typically considers only muscle mass at the levels of lumbar vertebra 3 and 4 (L3/L4) via skeletal muscle index (SMI) acquired using computed tomography (CT) (Kim et al., 2017), without the simultaneous assessment of muscle function or muscle quality. This approach is in contrast to the updated guidelines from the European Working Group on Sarcopenia in Older People (EWGSOP), which recommend initial assessment of muscle function before muscle mass as confirmation of sarcopenia status (Cruz‐Jentoft et al., 2019). Crucially, it is known, at least in primary sarcopenia, that muscle function is better at predicting adverse outcomes than muscle mass (Schaap et al., 2018). Therefore, there is a risk of missing key information influencing patient assessment by considering muscle mass alone.

Over the last two decades, CT‐derived SMI at L3 have been utilised in chronic disease states as indirect measures of whole body muscle mass, largely due to the reported correlation with whole body fat free mass and its association with mortality risk (Carey et al., 2017; Giusto et al., 2015). However, more recent evidence has questioned whether alternative muscle groups, such as the quadriceps, have a greater relevance to the performance of functional tasks. Indeed, these muscle groups may be more appropriate for the assessment of muscle health due to an increased sensitivity to change in disease states associated with sarcopenia (Cruz‐Jentoft et al., 2019; Wilhelm et al., 2014). Previous work by Tandon and colleagues investigated quadriceps muscle thickness via ultrasound as a surrogate measure to predict sarcopenia status in ESLD (determined by MRI/CT derived L3/4 SMI) (Tandon et al., 2016). The authors demonstrated that quadriceps muscle thickness could identify patients with sarcopenia; however, the study did not consider the impact on lower limb function or muscle quality. Therefore, a deep phenotypic investigation of the quadriceps muscle group in patients with ESLD remains elusive. In addition, little work has compared imaging modalities (i.e., ultrasound and MRI) for the assessment of quadriceps muscle mass and thus the validity of bedside ultrasound methods for lower limbs in ESLD remains ambiguous. Ultrasound‐based approaches would enable the relatively cheap and accessible assessment of lower limb mass within this patient group, enabling the identification of muscle loss and/or sarcopenia and in turn the facilitation of therapeutic interventions.

Aside from muscle mass, the investigation of muscle quality is also imperative to truly understand underlying muscle health and pathology (McGregor et al., 2014). Myosteatosis, that is, the infiltration of fat into skeletal muscle, is a key measure of muscle quality and can negatively affect muscle function (Biltz et al., 2020; Linge et al., 2021; Zamboni et al., 2019). Whilst previous studies have shown that myosteatosis occurs in patients with cirrhosis (Bhanji et al., 2018; Montano‐Loza et al., 2016), this has been via CT of the L3/L4 muscle groups, and not within the lower limbs. Nonetheless, a recent UK Biobank study suggested that adverse muscle composition of the thigh was associated with poorer function and metabolic comorbidities in patients with non‐alcoholic fatty liver disease (Linge et al., 2021). Aside from myosteatosis, other indirect measures of muscle quality can describe the force generating capacity of the muscle, such as specific force (Alcazar et al., 2018) or effective physiological cross‐sectional area (PCSA_eff_), with the latter providing an index of total contractile material (i.e., sarcomeres and cross bridges) as well as considering force transmission to the tendon (Maden‐Wilkinson et al., 2020). Regardless of the variable investigated, muscle quality will impact upon muscle health and as such should be considered alongside muscle mass.

Therefore, the main aim of this cross‐sectional study was to complete a deep phenotyping of muscle mass, quality and function in patients with ESLD and compare this to a healthy age‐ and sex‐matched control group. In addition, we aimed to investigate differences between upper, lower and trunk muscle groups with respect to muscle mass and function. As a secondary aim, the comparison between bedside ultrasonography and MRI imaging modalities of muscle mass would be investigated.

## 2 METHODS

### 2.1 Study population

Patients with ESLD were recruited from the liver transplant waiting list outpatient clinic at the Queen Elizabeth University Hospital Birmingham (UK) between 2019 and 2020; however, the presence of hepatocellular carcinoma was an exclusion criteria. The ESLD specific cohort was a component of the prospective observational study, the Evaluation of Sarcopenia in Inflammatory Disease (ESCID) (Dhaliwal et al., 2021). An additional healthy age‐ and sex‐matched control cohort was recruited. The healthy control (HC) group had no medical co‐morbidities, were not on medications and were deemed recreationally active (i.e., did not partake in any structured exercise). The study was approved by the Health Research Authority–West Midlands Solihull Research Ethics Service Committee Authority (REC reference: 18/WM/0167) and the study has been performed in accordance with the ethical standards laid down in the 1964 *Declaration of Helsinki* and its later amendments. All patients provided written informed consent. (ClinicalTrials.gov Identifier: NCT04734496).

### 2.2 Data collection

Demographics were collated for all participants, including weight, height, body mass index (BMI) and in the case of ESLD estimated dry weight and BMI were calculated (Table 1). In patients with ESLD, the disease type, severity (Child–Pugh score, model for end‐stage liver disease (MELD), UK model for end‐stage liver disease (UKELD)) and co‐morbidities were collected. All participants underwent the following investigations by certified members of the study team (J.Q., A.D., F.W.).

**TABLE 1:** Demographic and disease specific data for patients with end stage liver disease (ESLD) and healthy control (HC) groups.

	ESLD	HC	*P*
Demographics (mean ± SD)			
* n*	39	18	
Age (years)	55.0 ± 10.5	49.7 ± 14.9	NS
Males/females	24/15	11/7	
Height (m)	1.71 ± 0.12	1.71 ± 0.07	NS
Weight (kg)	90.8 ± 21.6	73.0 ± 12.2	*P* < 0.01
Ascites adjusted dry weight (kg)	86.1 ± 20.0	73.0 ± 12.2	*P* < 0.05
BMI (kg/m^2^)	30.1 ± 6.5	24.5 ± 3.4	*P* < 0.01
Dry weight BMI (kg/m^2^)	28.8 ± 6.3	24.5 ± 3.4	*P* < 0.05
Disease type (*n*, %)			
Alcoholic related liver disease	19 (48.7)	—	
Primary sclerosing cholangitis	9 (23.0)	—	
Primary biliary cholangitis	4 (10.2)	—	
Non‐alcoholic fatty liver disease	5 (12.8)	—	
Other	2 (5.2)	—	
Disease severity (median, IQR)			
MELD	11.0 (5)	—	
UKELD	52.0 (4)	—	
Child Pugh score	8.0 (3)	—	
Complications of ESLD (*n*, %)			
Hepatic encephalopathy	21 (53.8)	—	
Portal hypertension	21 (53.8)	—	
Ascites	29 (74.4)	—	
Diuretics	24 (61.5)	—	
Large volume paracentesis	7 (17.9)	—	
Spontaneous bacterial peritonitis	10 (25.6)	—	
Primary prophylaxis	17 (43.6)	—	
TIPPS	0	—	
Portal vein thrombosis	5 (12.8)	—	
Comorbidities, (*n*, %)			
Cardiovascular disease	4 (10.3)	0	
Hypertension	12 (30.8)	0	
COPD	5 (12.8)	0	
Diabetes mellitus	11 (28.2)	0	
Insulin dependent	8 (20.5)	0	
Hypercholesterolaemia	0	0	
Chronic kidney disease	7 (17.9)	0	
Blood analyses (median, IQR)			
HBA1C	33.5 (18.4)	34.5 (6)	N/S
Platelets	92 (60)	237 (61.3)	*P* < 0.0001
WCC	4.3 (3.1)	4.9 (1.2)	N/S
INR	1.2 (0.3)	1.0 (0.1)	*P* < 0.0001
Urea	5.4 (4.2)	5.1 (1.2)	N/S
Sodium	137 (5)	140 (2.2)	N/S
Creatinine	73 (36)	81 (20)	N/S
Bilirubin	35 (30)	12 (8)	*P* < 0.0001
Albumin	35 (9)	42.5 (5.2)	*P* < 0.0001
ALT	30 (33)	18 (6)	*P* < 0.01
ALP	167 (165)	71.5 (18.2)	*P* < 0.0001

Statistical significance between groups assessed via unpaired Student's *t*‐test or Mann–Whitney test. Values presented are means ± SD or median (IQR) depending upon parametric/non‐parametric data respectively. Dry weight was determined by subtracting the percentage of fluid based on clinical examination for mild (5%), moderate (10%), and severe (15%) ascites and/or mild (5%) and moderate (10%) peripheral oedema. ALP, alkaline phosphatase; ALT, alanine aminotransferase; COPD, chronic obstructive pulmonary disease; MELD, model for end‐stage liver disease; TIPPS, trans‐jugular intrahepatic porto‐systemic shunt; UKELD, UK model for end‐stage liver disease; WCC, white cell count.

### 2.3 Mid arm muscle circumference

Mid arm circumference (MAC) and triceps skin fold (TSF) thickness were measured (Dhaliwal et al., 2021) in order to calculate mid arm muscle circumference (MAMC):

(1) $$\mathrm{MAMC}\;=\;\mathrm{MAC}-\left[\pi \times \left(\frac{\mathrm{TSF}}{10}\right)\right]$$

### 2.4 Quadricep muscle ultrasonography

**FIGURE 1:**
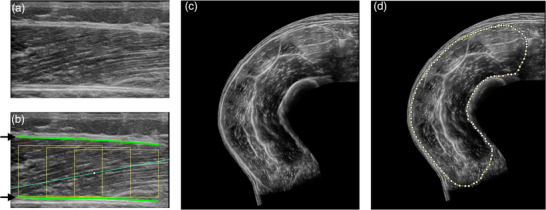
Illustrative images for the analysis of vastus lateralis muscle thickness (a, b) and quadriceps ACSA via ultrasound (c, d), taken from HC participant. (a) Sagittal scan of the vastus lateralis. (b) Semi‐automated software analysis. The superficial and deeper aponeuroses are identified by black arrows and green lines. The diagonal blue line represents the median fascicle architecture. (c) An EFOV ultrasound ACSA scan of the quadriceps. (d) Manual analysis and quantification of quadriceps ACSA.

Sagittal ultrasound images of the vastus lateralis (VL) were obtained as previously described (Quinlan et al., 2021), using a portable ultrasound instrument (MyLab Alpha, Esaote, Genoa, Italy) attached with a 3–13‐MHz, 4.7 cm linear array transducer (SL1543, Esaote). Measurements were performed on the dominant leg with the participant lying supine, following a 10‐min rest period. Scans were acquired at 50% of the distance between the greater trochanter and midpoint of the patella. In addition, the mid sagittal axis of the VL was obtained (identified as the mid‐point between the medial and lateral borders of the muscle, as assessed by ultrasound). Images were analysed using a semi‐automated Fiji macro tool (Simple Muscle Architecture Analysis, V1.7; Seynnes & Cronin, 2020) which allowed for the estimation of VL muscle thickness, fascicle length and pennation angle (Figure 1). Analysis was completed in triplicate and mean average values were used. In addition to the above, an extended field of view (EFOV) ultrasound technique was used to acquire a full quadriceps anatomical CSA (ACSA) at 50% femur length mark of the dominant leg (Figure 1) as previously described (Monti et al., 2020). Following acquisition, scans were manually assessed for quadriceps ACSA via offline software (Fiji, v2.1.0).

### 2.5 Magnetic resonance imaging of muscle

#### 2.5.1 L3 Skeletal muscle index

Images were collected via a 3 T Cobalt MRI scanner with T1‐weighted turbo spin echo sequence with repetition time 600 ms, echo time 15.2 ms, field of view 512 × 512 mm and 1 cm slice thickness with no slice gap. Muscle CSA at the L3 level was manually segmented by a single investigator via Horos software (version 3.3.6) similar to previous work (Quinlan et al., 2022). Muscles at the L3 region included the psoas, erector spinae, quadratus lumborum, transversus abdominus, external and internal obliques and rectus abdominus. Consequently, L3 SMI was calculated by normalising L3 muscle CSA to height squared of each participant.

#### 2.5.2 Quadriceps muscle volume

Manual segmentation of the dominant leg quadriceps was completed by a single investigator via Horos (version 3.3.6). The calculation of quadriceps muscle volume utilised on average seven ACSA slices with 4 cm interslice thickness from a restricted quadriceps region of interest (ROI) (Quinlan et al., 2022). This restricted ROI omitted proximal and distal extremes of the quadriceps to increase accuracy (McGlory et al., 2019). The proximal limit was identified as the appearance of the lesser trochanter and the distal limit as 20% above the proximal aspect of the patella (Quinlan et al., 2022). Any sections which were identified as adipose or non‐contractile tissue were excluded from muscle ACSA analysis and muscle volume was estimated:

(2) $$\begin{array}{ccc} \mathrm{MV} & & =\sum \left(\left(\frac{1}{3}\times \mathrm{slice}\;\mathrm{thickness}\right)\right.\\ & & \left.\times \;\left[\mathrm{CS}A_{n}+\sqrt{\left(\mathrm{CS}A_{n}\times \;\mathrm{CS}A_{n+1}\right)}+\mathrm{CS}A_{n+1}\right]\right) \end{array}$$

where MV is muscle volume (MV) and both the current (CSA*n*) and sequential (CSA*n*+1) ACSA are required for the estimation. Quadriceps muscle volume was normalised to height squared for each individual so that muscle volume is presented as quadriceps volume index.

#### 2.5.3 Quadriceps Mid‐ACSA

In addition to the ACSA values obtained for muscle volume estimation, quadriceps ACSA at 50% of femur length (distance between greater trochanter and medial patella), was also calculated for the dominant leg. An oil capsule was fixed at this 50% point prior to MRI, enabling identification. Post‐acquisition, manual segmentation of this identified slice was completed and reported as mid ACSA.

#### 2.5.4 Vastus lateralis muscle volume and physiological cross‐sectional area

VL muscle volume was estimated in similar fashion to quadriceps muscle volume, in order to calculate VL physiological cross‐sectional area (PCSA). However, in contrast to the quadriceps muscle volume, ACSA slices are obtained across the full length of the VL rather than the restricted ROI. VL PCSA was then calculated as VL muscle volume divided by VL fascicle length as previously reported (Maden‐Wilkinson et al., 2020). In order to correct for force transmission to the tendon and provide the most accurate measure of muscular torque production, effective PCSA (PCSA_eff_), was calculated by multiplying PCSA by the cosine of VL pennation angle (Maden‐Wilkinson et al., 2020).

#### 2.5.5 Intermuscular adipose tissue

Intermuscular adipose tissue (IMAT) was estimated via the two‐point Dixon sequence method (Ogawa et al., 2020) with offline analysis with Horos software (version 3.3.6). IMAT was calculated on the mid ACSA as previously identified. Specifically, manual segmentation of the quadriceps ACSA was completed on both the ‘fat only’ and ‘water only’ fractions and the mean signal intensity of the quadriceps ROI was calculated for each fraction. IMAT percentage was then calculated and the data presented herein are generated from the dominant leg:

(3)

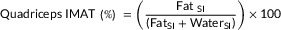

where SI is signal intensity (SI) of fat only and water only fractions.

### 2.6 Muscle strength and function

#### 2.6.1 Chair stands

Chair stands were completed as a component of the Short Physical Performance Battery and occurred prior to peak isokinetic assessment to prevent any carry over fatigue. Participants were asked to complete five sit to stands in the quickest time possible, ensuring they stood up and sat back fully on each repetition. The time taken to complete five full sit to stands was recorded.

#### 2.6.2 Handgrip strength

Peak dominant handgrip strength (HGS) was assessed as the highest value of three attempts via a hand grip dynamometer (Takei, 5401 GRIP‐D).

#### 2.6.3 Isokinetic quadriceps strength

Measurements of unilateral peak isokinetic torque for the knee extensors (quadriceps) was conducted on the Biodex Medical System 3 (Biodex Medical Systems, Shirley, NY, USA). The assessment protocol consisted of five consecutive maximal isokinetic leg extension contractions (60 deg/s) of the non‐dominant leg. The non‐dominant limb had to be utilised as muscle biopsies were collected from the dominant leg prior to this assessment as part of the ESCID study (Dhaliwal et al., 2021). ESLD participants underwent a familiarisation session within 2 weeks prior to the ESCID study, whereby we observed no difference in peak torque between dominant and non‐dominant leg, thus validating the use of the non‐dominant limb herein (103 vs. 100 N m, respectively). Peak isokinetic torque was defined as the highest recorded torque value during the five completed contractions.

#### 2.6.4 Specific force

Specific force (i.e., force per unit area of muscle) was calculated as the peak torque divided by mid quadriceps ACSA (identified by the oil capsule):

(4) $$\mathrm{Specific}\;\mathrm{force}\;=\frac{\mathrm{Isokinetic}\;\mathrm{torque}\;}{\mathrm{Quadriceps}\;\mathrm{ACSA}\;\times \;\left(1\;-\;\mathrm{IMAT}\;\%\right)}\;$$

This value was calculated for the non‐dominant limb as per the reasons previously explained and utilised the same methodology described above. For the calculation of specific force, quadriceps ACSA was normalised to IMAT percentage, such that a more accurate estimation of contractile tissue was considered.

### 2.7 Physical activity

In order to assess habitual physical activity, participants were provided with a wrist worn GENEActiv (Activinsights, Cambridge, UK), which was worn for a minimum of 3 days and maximum of 14 days prior to the visit. Data were excluded if post‐calibration error was greater than 0.01 g, less than three valid days (≥16 h wear) were obtained, or wear data were not present for each 15 min period of the 24 h cycle. As such, data were only available for 30/39 ESLD patients and 17/18 HC participants. Extracted accelerometer files were processed and analysed with an open‐source R package, GGIR (version 2.5‐0, http://cran.r‐project.org) (Rowlands et al., 2016). The average acceleration of movement, a proxy for total activity, was calculated for each valid day and consequently averaged across all valid days. In addition, time spent above the moderate–vigorous physical activity (MVPA) threshold (>100 m*g*) was calculated for each valid day, and as above, averaged across all valid days.

### 2.8 Statistical analysis

All data were analysed utilising GraphPad Prism software, version 9 (GraphPad Software, La Jolla, CA, USA). Data were checked for normal distribution with the D'Agostino and Pearson test and presented as means ± standard deviation when normally distributed and medians (IQR) when non‐normally distributed. Comparisons between groups were completed via either an unpaired Student's *t*‐test or unpaired Mann–Whitney test. Finally, correlations were assessed via Pearson's *r* statistical test and adjusted if non‐parametric. Cohen's *d* was used to calculate the effect size, where *d* = 0.2, 0.5 and 0.8 indicate a small, medium and large effect, respectively. The level of significance was set at *P* < 0.05 throughout.

## 3 RESULTS

### 3.1 Participant characteristics

A total of 57 individuals were studied, thirty‐nine were patients with ESLD on the UK's national transplant waiting list, with a mean age 55.0 years and male predominance (61.5%). The most common aetiology of ESLD was alcohol‐related liver disease (19/39; 48.7%), followed by chronic biliary diseases (13/39; 33.3%) (Table 1). The median Child–Pugh score was 8/15, with evident ascites and hepatic encephalopathy in 74% and 53%, respectively. In total, there were 18 age‐ and sex‐matched HC (mean age of 49.7 years and 61.1% male dominance), whose demographic and clinical parameters are highlighted in Table 1.

### 3.2 Measures of muscle mass

#### 3.2.1 ESLD versus HC

**FIGURE 2:**
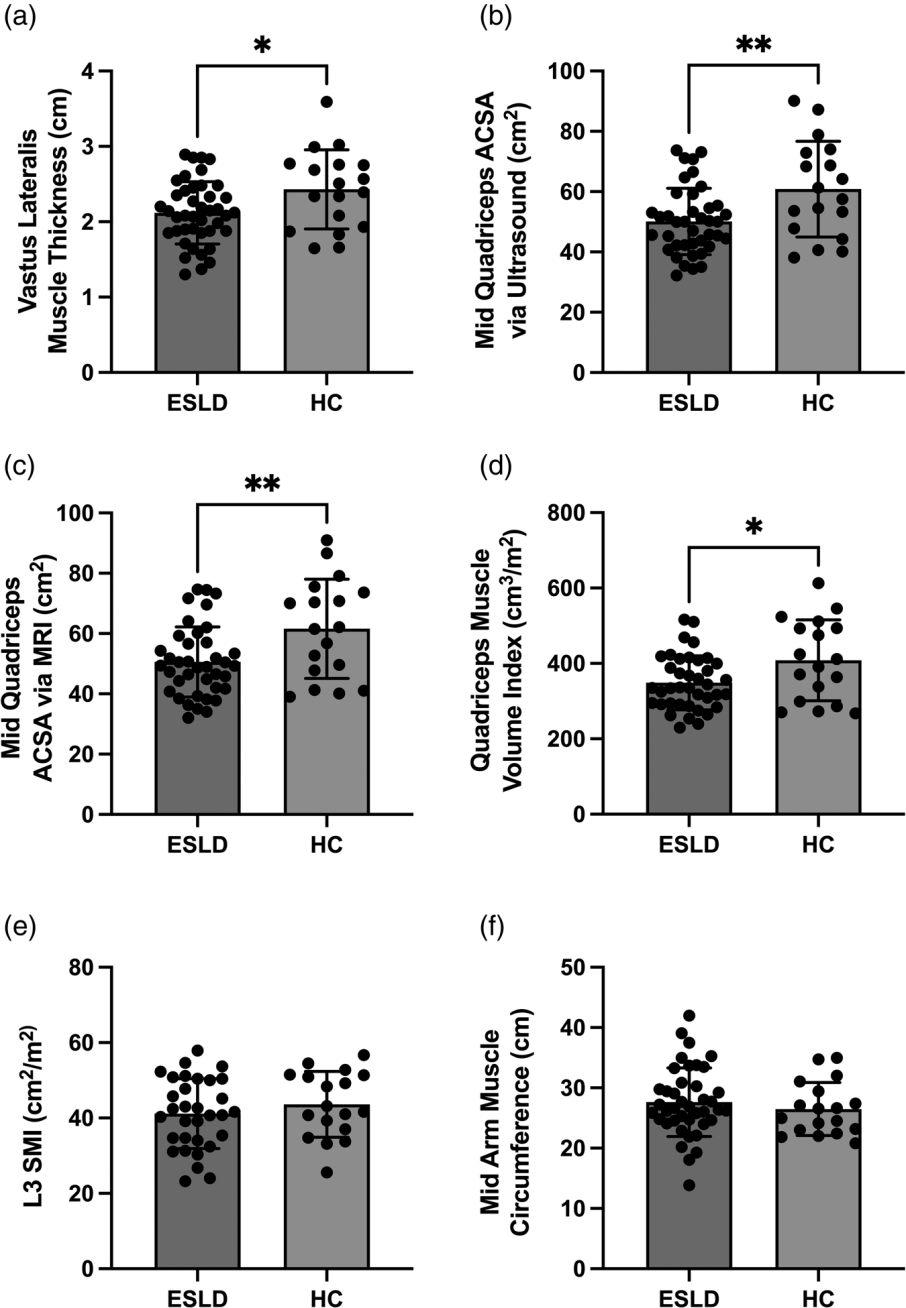
Differences in muscle mass in lower limb via ultrasound (a,b), lower limb via MRI (c,d), trunk (e) and upper limb (f) between patients with end stage liver disease (ESLD) and healthy age/sex matched control participants (HC). Data are expressed as grouped mean average with individual data points. Significance between groups identified as *P < 0.05 and **P < 0.01, *n* = 32 for ESLD in (e).

Overall, quadriceps muscle mass was significantly lower in patients with ESLD compared to HC. This was evidence by reductions in vastus lateralis muscle thickness as measured via B‐mode ultrasound (2.11 ± 0.4 cm vs. 2.43 ± 0.5 cm, *P* < 0.05, *d* = 0.69 Figure 2a) and mid quadriceps ACSA as measured via EFOV ultrasound (50.1 ± 11.0 vs. 60.9 ± 15.8 cm^2^, *P* < 0.01, *d* = 0.8 Figure 2b). These reductions were also apparent via MRI muscle measures, with both MRI mid quadriceps ACSA (50.5 ± 11.5 vs. 61.6 ± 16.4 cm^2^, *P* < 0.01, *d* = 0.79 Figure 2c) and MRI quadriceps volume index (346 ± 72 vs. 410 ± 110 cm^3^/m^2^, *P* < 0.05, *d* = 0.7 Figure 2d). However, these difference in muscle mass were not evident when considering MAMC (27.6 ± 5.6 vs. 26.4 ± 4.3 cm, NS, *d* = 0.24) or in the MRI measure of L3 SMI (41.2 ± 9.2 vs. 43.6 ± 8.7 cm^3^/m^2^, NS, *d* = 0.26) (Figure 2e,f, respectively).

#### 3.2.2 Comparison of ultrasound and MRI quadriceps muscle mass

**FIGURE 3:**
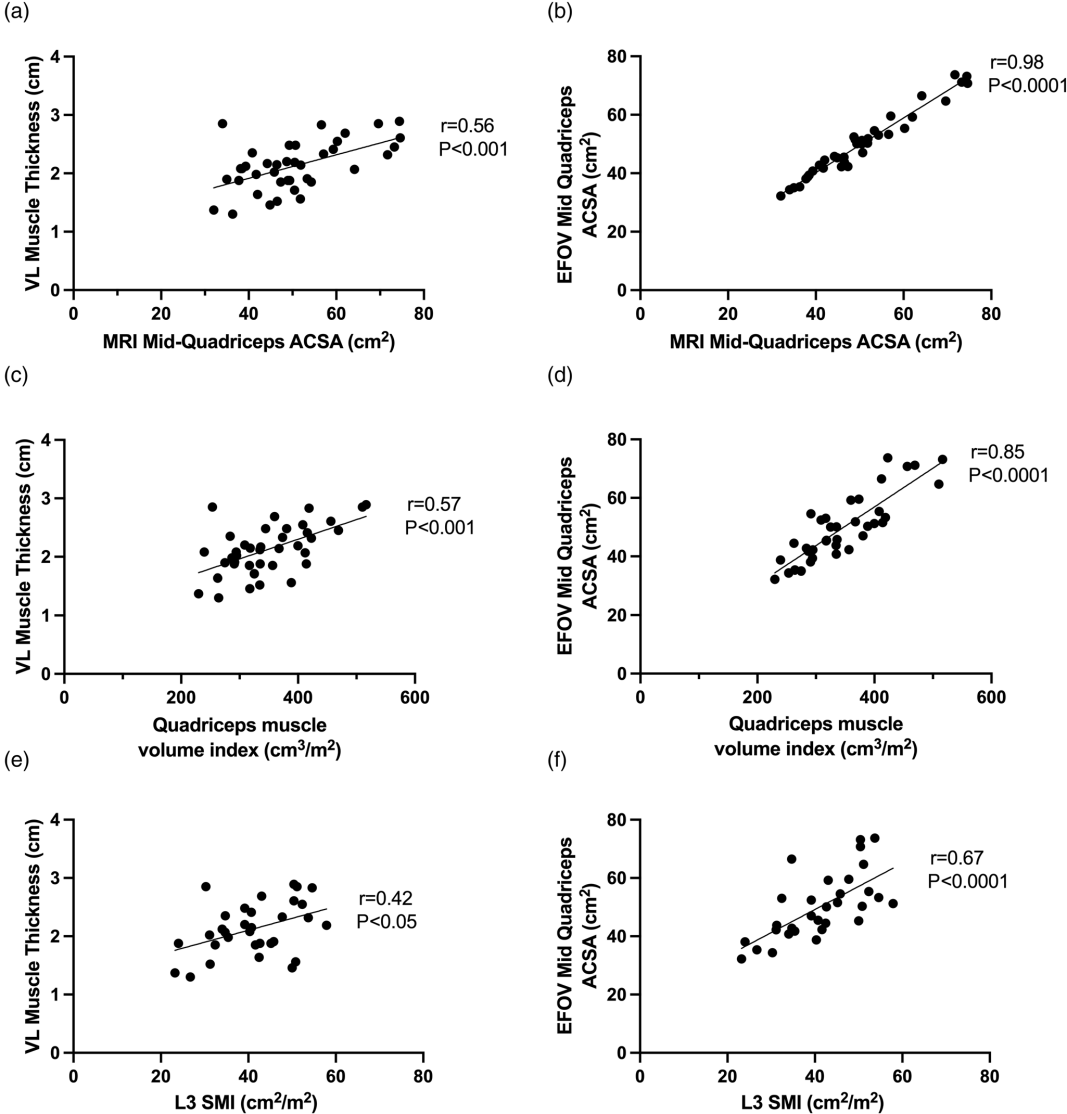
Correlations between ultrasound measures and MRI measures of muscle mass in end stage liver disease. (a,c,e) Correlations between vastus lateralis muscle thickness via ultrasound and MRI measures of quadriceps mass (a,c) and L3 SMI (e). (b,d,f) Correlations between quadriceps ACSA obtained via extended field of view (EFOV) ultrasound and MRI measures of quadriceps mass (b,d) and L3 SMI (f). *n* = 39 for (a–d) and *n* = 32 for (e,f).

We compared measures of quadriceps muscle mass as obtained via ultrasound to gold standard MRI measures, including measures of quadriceps ACSA, quadriceps volume index and trunk measure of L3 SMI. Our data show that ultrasound measures of VL muscle thickness correlate well to MRI measures of quadriceps ACSA (*r* = 0.56, *P* < 0.001), MRI quadriceps volume index (*r* = 0.57, *P* < 0.001) and MRI L3 SMI (*r* = 0.42, *P* < 0.05) (Figure 3a,c,e). The EFOV ultrasound approach also showed excellent degrees of correlation to MRI measures, including quadriceps ACSA (*r* = 0.98, *P* < 0.0001), MRI quadriceps volume index (*r* = 0.85, *P* < 0.0001) and MRI L3 SMI (*r* = 0.67, *P* < 0.0001) (Figure 3b,d,f).

### 3.3 Muscle strength and function

**FIGURE 4:**
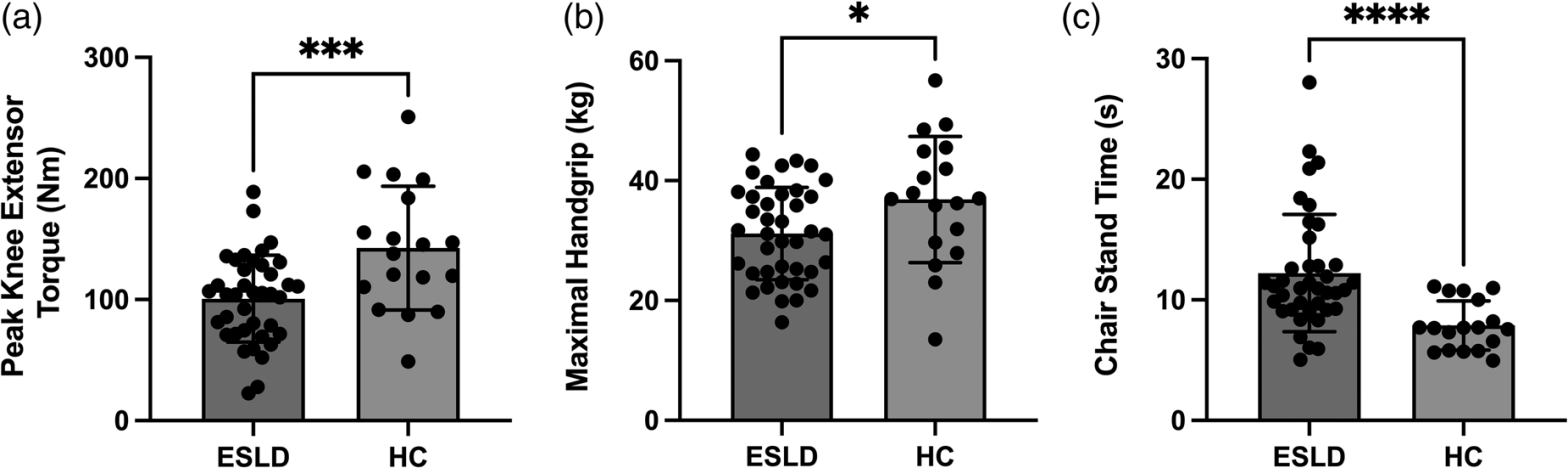
Differences in physical function (a,b) and performance (c) between patients with end stage liver disease (ESLD) and healthy age matched control participants (HC). Data are expressed as grouped mean average with individual data points. Significance between groups identified as *P < 0.05,***P < 0.001, ****P < 0.0001.

**FIGURE 5:**
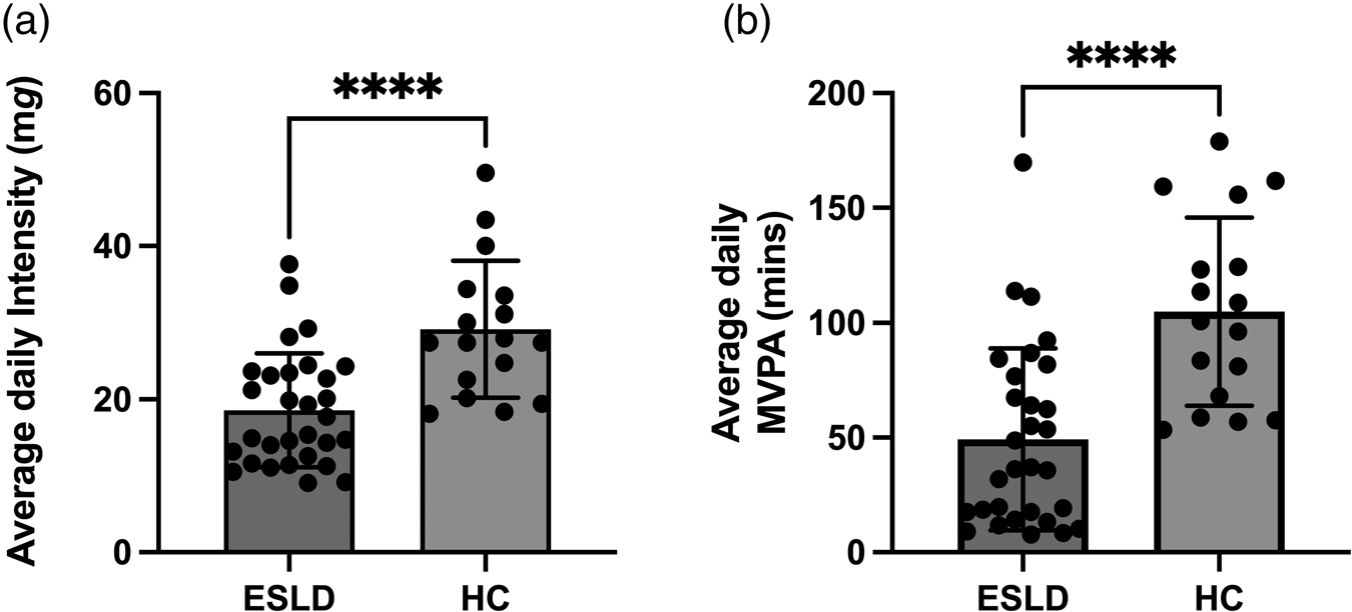
Differences in physical activity (a) and moderate–vigorous physical activity (MVPA) (b) between patients with end stage liver disease (ESLD) and healthy age/sex‐matched control participants (HC). Data are expressed as grouped mean average with individual data points. Significance between groups identified as **** *P* < 0.0001, *n* = 30 for ESLD and *n* = 17 for HC.

Regardless of assessment modality, all measures of upper and lower limb muscle function and/or strength demonstrated that patients with ESLD were significantly impaired compared to HC. This was the case for lower limb strength and function with lower peak isokinetic leg extensor torque (99.5 ± 35 vs. 142.5 ± 51 N m, *P* < 0.001, *d* = 1.0, Figure 4a) and prolonged chair stand times (10.9 (3.7) vs. 7.6 (4.4) s, *P* < 0.0001, *d* = 1.26, Figure 4c). Upper limb strength was also significantly lower in ESLD compared to HC with reduced peak handgrip strength (31.1 (12.9) vs. 36.9 (15.8) kg, *P* < 0.05, *d* = 0.63, Figure 4b). Our data also show that quadriceps muscle mass correlated to peak isokinetic knee extensor torque when mid‐ACSA (*r* = 0.68, *P* < 0.0001) and quadriceps volume index (*r* = 0.66, *P* < 0.0001) was considered (not shown). Finally, in addition to reductions in strength and function, both total daily physical activity (18.6 ± 7.4 vs. 29.1 ± 8.9 mg, *P* < 0.0001, *d* = 1.29 Figure 5a) and average daily MVPA (36.6 (61.3) vs. 100.7 (76.45) min, *P* < 0.0001, *d* = 1.37, Figure 5b) were significantly lower in patients with ESLD compared to HC.

### 3.4 Muscle quality

**FIGURE 6:**
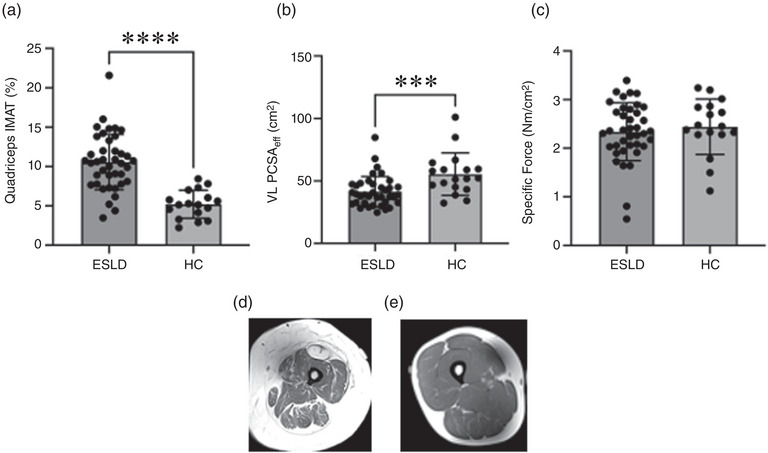
(a–c) Differences in quadriceps muscle quality between patients with end stage liver disease (ESLD) and healthy age matched control participants (HC). (d,e) Representative quadriceps MRI scans for ESLD and HC respectively. Data are expressed as grouped mean average with individual data points. Significance between groups identified as ***P < 0.001 and ****P < 0.0001. IMAT, intermuscular adipose tissue; VL, vastus lateralis; PCSA_eff_, effective physiological cross‐sectional area.

In general muscle quality of the quadriceps was reduced in ESLD compared to HC. The ESLD patient group had approximately double the amount of myosteatosis in comparison to HC, as measured by quadriceps IMAT via MRI (10.5 ± 3.5% vs. 5.2 ± 1.7%, *P* < 0.01, *d* = 2.04, Figure 6a). In addition, the quality of the VL was reduced in the form of altered muscle architecture (measured via ultrasound) with lower pennation angle of the VL in ESLD compared to HC (12.5 ± 3.0 vs. 16.2 ± 3.9 deg, *P* < 0.001, *d* = 1.07); however, fascicle length did not differ. In line with the above, PCSA_eff_ of the VL was significantly higher in HC compared to ESLD, reflecting a greater muscle quality within the VL (38.7 (14.8) vs. 52.0 (16.1) cm^2^, *d* = 0.96, *P* < 0.001, Figure 6b). However, when specific force was considered, we observed no difference between the two groups (Figure 6c).

## 4 DISCUSSION

This study extensively characterised muscle health in the form of mass, function and quality within upper, lower and trunk muscle groups in patients with ESLD. We highlight that in comparison to age‐ and sex‐matched healthy control participants, patients with ESLD have significantly compromised muscle mass, poorer muscle quality and consequently reduced muscle function of the lower limbs. However, this reduction in muscle mass was lower in the conventional mass measures at trunk (L3 SMI) or upper limb (MAMC), suggesting that the lower limbs may be more susceptible to detrimental muscle alterations in ESLD. The concept of a more preserved upper limb muscle mass and greater muscle loss in lower limb has previously been reported in primary sarcopenia (Janssen et al., 2000), but not in ESLD. It is believed that the quadriceps possess greater sensitivity to change in disease states associated with sarcopenia compared to upper limbs which is consistent with our data, likely due to their role in daily tasks (Cruz‐Jentoft et al., 2019; Wilhelm et al., 2014).

Our data may have implications for the assessment of sarcopenia and/or muscle loss in patients with ESLD. Indeed, the current gold standard approach to measure muscle mass within the hepatology field is L3 SMI (via MRI or CT) (Lai et al., 2021; Williams et al., 2021); however we herein show that reductions in muscle mass may be greater within the quadriceps (∼−17% quadriceps ACSA vs. HC, *d* = 0.8) compared to that of L3 SMI (∼−5% vs. HC, *d* = 0.26). As such, the use of L3 SMI may lead to an underappreciation of the reductions in muscle mass present in ESLD. It has previously been stated that the use of L3 SMI is beneficial due to the relative independence from activity levels (Dasarathy & Merli, 2016). Contrary to muscles at L3, the quadriceps have high functional relevance and hence are likely impacted by reduced physical activity levels seen in ESLD, in turn further compounding deficits in muscle mass that would not be observed by considering L3 muscle mass. MAMC, a commonly used clinical assessment for muscle mass and nutritional status, followed a similar trend to that of L3 SMI, that is, no difference to HC. These deficits in quadriceps muscle mass (−13% to −17% depending on variable) are equivalent to the muscle loss observed in 42–56 days of bedrest in healthy individuals or ∼10 days in an intensive treatment unit (Hardy et al., 2022). Further, age‐related sarcopenia is associated with a 0.5–1% annual loss of muscle mass (Mitchell et al., 2012; Volpi et al., 2004); thus the deficits seen in ESLD would equate to approximately 10–15 years of additional ageing. We therefore suggest that to truly appreciate reductions in muscle mass in ESLD, a greater emphasis should be placed upon lower limb muscle groups, such as the quadriceps.

In order to assist with the assessment of quadriceps muscle mass and in turn aid sarcopenia assessment in ESLD; the validation of cheaper and more accessible bedside imaging methodologies, such as ultrasonography, is essential. We show that both VL muscle thickness and quadriceps ACSA via EFOV ultrasound have good to excellent correlation with MRI‐obtained measures of quadriceps mass and L3 SMI (Figure 3). The use of VL muscle thickness is regularly employed within primary sarcopenia and ageing research (Franchi et al., 2018), but this inevitably only considers one muscular component of the quadriceps. Unsurprisingly, more robust correlations were seen between ultrasound and MRI when the EFOV ultrasound approach (Figure 1) was utilised to estimate full quadriceps ACSA (Figure 3). Like VL muscle thickness, the use of EFOV ultrasound is well reported in the literature, but often only applied within healthy, non‐diseased individuals (Sarto et al., 2021) and is rarely utilised within clinical practice. Nonetheless we show excellent correlation between quadriceps ACSA generated from EFOV ultrasound and MRI measures of both quadriceps specific measures (i.e., ACSA and volume) and L3 SMI measures. As such, our data provide evidence for the use of bedside ultrasound approaches for a single time point assessment of lower limb muscle mass in ESLD.

The loss of quadriceps muscle mass described above likely explains the reduced lower limb muscle function observed in ESLD. This concept is supported by the evident correlation between quadriceps muscle mass and peak knee extensor torque, as well as a lack of difference in specific force (i.e., peak force normalised to muscle size and IMAT content). Despite the latter result being somewhat surprising, it would suggest the ability of the contractile tissue to produce force is maintained in ESLD, at least in the quadriceps, in comparison to an age‐matched control group. Indeed, a strong correlation between quadriceps muscle mass and isokinetic knee extensor strength (*r* = 0.68) reinforces the concept that the difference in maximal muscle strength is predominately due to reduced muscle mass rather than differences in the intrinsic force‐generating capacity of the tissue. This highlights the importance of considering quadriceps muscle mass as an outcome measure during intervention. Nonetheless, aside from changes in muscle mass, we did also observe differences in VL pennation angle and VL PCSA_eff_ (lower in the ESLD). A lower VL pennation angle is also seen as the result of ageing (Quinlan et al., 2021) and typically reflects a lower amount of contractile tissue along the deeper aponeurosis (Franchi et al., 2017) and hence may negatively impact force output as well as negatively impacting force transmission (Narici et al., 2016). In order to account for this effect, values of PCSA_eff_ are often utilised, which accounts for both muscle volume and muscle architecture, i.e., both pennation angle and fascicle length. PCSA_eff_ therefore considers the ability of the muscle to both produce and transmit muscle force to the joint (Maden‐Wilkinson et al., 2020). As aforementioned VL PCSA_eff_ was lower in the ESLD group and as such may further explain the reduction in peak force output, aside from muscle mass alone. Muscle architecture and PCSA_eff_ were only assessed in the VL, and so may not apply to the full quadriceps. However, these results do provide further evidence that many negative alterations exist within lower limb muscles in ESLD.

Another important component of muscle quality is fat infiltration within the muscle, that is, myosteatosis. We observed that relative IMAT within the quadriceps of those with ESLD was higher than the HC group. The presence of IMAT is known to negatively impact force production and consequently muscle function (Biltz et al., 2020; Buford et al., 2012; Tuttle et al., 2012), and as such, the higher levels seen in ESLD may partially explain the differences in muscle force observed. In addition to impacting muscle strength, the presence of high IMAT is also associated with metabolic dysfunction such as insulin resistance and inflammation, which would only further compound poor muscle health (Addison et al., 2014). Thus, it is likely that the reduction in peak force production observed in ESLD (in the lower limbs) is likely the result of reductions in both absolute muscle mass and poor muscle quality.

Aside from peak force production, physical performance (chair stand time) was also negatively impacted in ESLD. Interestingly we observed a large range of times obtained in the ESLD group (5.0–23.0 s), suggesting that this real‐world functional assessment may be discriminating differences in functional performance. Indeed, previous work has demonstrated that chair stand score is a strong predictor of transplant waiting list mortality (Lai et al., 2017). However, intrinsic ESLD factors not present in the HC group such as the discomfort caused by ascites (present in ∼75% of ESLD group) may also influence the observed differences between groups (Alcazar et al., 2022). Nonetheless, upper limb strength was also lower in ESLD compared to HC, demonstrating that declines in muscle function appear whole body wide and irrespective of assessment modality, although, reduction in lower limb muscle strength appeared to be greater than that of upper limb strength (approximately −29% and −15% for knee extensor torque and handgrip respectively). Similar to the disparity in muscle mass loss, functional decline is believed to be greater in lower limb compared to upper limb in primary sarcopenia (Larsson et al., 2019). This finding is significant as there is a greater importance of lower limb strength in day‐to‐day tasks associated with independent living (Pasco et al., 2020; Reid & Fielding, 2012). While the underlying mechanisms of muscle mass and functional loss in ESLD may differ to that of primary sarcopenia, it is likely that some degree of overlap exists. Indeed, physical activity is known to be a key regulator of muscle mass, and it is particularly relevant within the lower limbs due to their use in day‐to‐day activities such as walking and climbing stairs (Breen & Phillips, 2011). We observed that both the intensity of habitual physical activity and daily MVPA time were reduced in the ESLD group compared to HC group (Figure 5), which may in part have exacerbated the reduced muscle mass observed in patients with ESLD. In addition to lifestyle factors, alterations to key catabolic molecular pathways (e.g., increased myostatin), impaired mitochondrial function, accelerated starvation, amino acid deprivation, chronic inflammation and hyperammonemia are all suggested to contribute to muscle loss in ESLD (Allen et al., 2020, 2022; Dasarathy & Merli, 2016). Ultimately, the above are known to negatively impact muscle protein turnover (i.e., reduced protein synthesis increased protein breakdown), which in turn may explain the reduced muscle mass observed herein. However, future work in this area is needed to truly elucidate these mechanisms.

We recognise there are several limitations to this study. Firstly, the study took a cross‐sectional approach to evaluate the effect of ESLD on muscle health by comparing to a separate healthy control group. Evaluating the effect of ESLD on sequential muscle health and different muscle compartments (upper and lower limb) at different time points will be key in order to understand natural progression and the impact of target therapies (i.e., exercise, nutrition and pharmaceuticals). We also acknowledge that the clinical sample size herein (ESLD *n* = 39) is relatively small, and due to the heterogeneity of ESLD, these findings should be explored on a larger scale before any definitive conclusions can be drawn. Nonetheless, our data provide the premise for future studies to consider a more comprehensive view of muscle health in ESLD. Finally, due to the Covid‐19 pandemic, the recruitment of the HC group was limited to University of Birmingham and Birmingham Hospital Trust staff members. Largely due to ‘work from home’ mandates within the UK during this recruitment phase, it is possible that habitual activity and hence muscle mass and function may have been negatively impacted in the HC group, thus reducing the difference between the two groups.

### 4.1 CONCLUSION

This study demonstrates that muscle functional deficits in ESLD (i.e., compared to age‐ and sex‐matched controls) were observed in both upper and lower limbs; however, greater differences were seen in lower limbs. This concept is also reflected in the differences in muscle mass, that is, greater differences observed in lower limbs compared to trunk and upper limb muscle groups. Whilst it is likely that reduced muscle mass can explain the reductions in muscle function, the reduction in muscle quality, that is, increased myosteatosis, is likely also a contributing factor. Collectively, our data suggest that muscle mass, quality and function should all be considered alongside one another when considering muscle health in ESLD. Finally, a greater consideration should be placed on muscle mass and quality within functionally relevant lower limb muscles, such as the quadriceps, due to their significant impact upon independence and hence patient quality of life in those with ESLD.

## AUTHOR CONTRIBUTIONS

Jonathan I. Quinlan, Amritpal Dhaliwal, Felicity Williams, Surabhi Choudhary, Leigh Breen, Janet M. Lord, Ahmed M. Elsharkawy, Matthew J. Armstrong, Carolyn A Greig: conception or design of the work; acquisition, analysis, or interpretation of data for the work; drafting of the work or revising it critically for important intellectual content; approved the final version of the manuscript; agree to be accountable for all aspects of the work in ensuring that questions related to the accuracy or integrity of any part of the work are appropriately investigated and resolved. Sophie L Allen, Alex Rowlands: acquisition, analysis, or interpretation of data for the work; drafting of the work or revising it critically for important intellectual content; approved the final version of the manuscript; agree to be accountable for all aspects of the work in ensuring that questions related to the accuracy or integrity of any part of the work are appropriately investigated and resolved. Gareth G Lavery: conception or design of the work; drafting of the work or revising it critically for important intellectual content; approved the final version of the manuscript; agree to be accountable for all aspects of the work in ensuring that questions related to the accuracy or integrity of any part of the work are appropriately investigated and resolved.

## CONFLICT OF INTEREST

The authors declare no conflict of interest.

## Data Availability

All individual data points are presented within the manuscript.

## References

[eph13373-bib-0001] Addison, O. , Marcus, R. L. , Lastayo, P. C. , & Ryan, A. S. (2014). Intermuscular fat: A review of the consequences and causes. International Journal of Endocrinology, 2014, 309570. 24527032 10.1155/2014/309570 PMC3910392

[eph13373-bib-0002] Alcazar, J. , Ara, I. , García‐García, F. J. , & Alegre, L. M. (2022). Number of chair stands should not be considered a muscle function measure, but a physical performance measure. What can we do then? Journal of Frailty & Aging, 11, 245–246. 35441206 10.14283/jfa.2021.50

[eph13373-bib-0003] Alcazar, J. , Rodriguez‐Lopez, C. , Ara, I. , Alfaro‐Acha, A. , Rodríguez‐Gómez, I. , Navarro‐Cruz, R. , Losa‐Reyna, J. , García‐García, F. J. , & Alegre, L. M. (2018). Force‐velocity profiling in older adults: An adequate tool for the management of functional trajectories with aging. Experimental Gerontology, 108, 1–6. 29567100 10.1016/j.exger.2018.03.015

[eph13373-bib-0004] Allen, S. L. , Quinlan, J. I. , Dhaliwal, A. , Armstrong, M. J. , Elsharkawy, A. M. , Greig, C. A. , Lord, J. M. , Lavery, G. G. , & Breen, L. (2020). Sarcopenia in chronic liver disease: Mechanisms and countermeasures. American Journal of Physiology. Gastrointestinal and Liver Physiology, 53, G241–G257. 10.1152/ajpgi.00373.2020 PMC8609568 33236953

[eph13373-bib-0005] Allen, S. L. , Seabright, A. P. , Quinlan, J. I. , Dhaliwal, A. , Williams, F. R. , Fine, N. H. F. , Hodson, D. J. , Armstrong, M. J. , Elsharkaway, A. M. , Greig, C. A. , Lai, Y. , Lord, J. M. , Lavery, G. G. , & Breen, L. (2022). The effect of ex vivo human serum from liver disease patients on cellular protein synthesis and growth. Cells, 11(7), 1098. 35406665 10.3390/cells11071098 PMC8997893

[eph13373-bib-0006] Bhanji, R. A. , Moctezuma‐Velazquez, C. , Duarte‐Rojo, A. , Ebadi, M. , Ghosh, S. , Rose, C. , & Montano‐Loza, A. J. (2018). Myosteatosis and sarcopenia are associated with hepatic encephalopathy in patients with cirrhosis. Hepatology International, 12(4), 377–386. 29881992 10.1007/s12072-018-9875-9

[eph13373-bib-0007] Biltz, N. K. , Collins, K. H. , Shen, K. C. , Schwartz, K. , Harris, C. A. , & Meyer, G. A. (2020). Infiltration of intramuscular adipose tissue impairs skeletal muscle contraction. The Journal of Physiology, 598(13), 2669–2683. 32358797 10.1113/JP279595 PMC8767374

[eph13373-bib-0008] Breen, L. , & Phillips, S. M (2011). Skeletal muscle protein metabolism in the elderly: Interventions to counteract the “anabolic resistance” of ageing. Nutrition and Metabolism, 8(1), 68. 21975196 10.1186/1743-7075-8-68 PMC3201893

[eph13373-bib-0009] Buford, T. W. , Lott, D. J. , Marzetti, E. , Wohlgemuth, S. E. , Vandenborne, K. , Pahor, M. , Leeuwenburgh, C. , & Manini, T. M. (2012). Age‐related differences in lower extremity tissue compartments and associations with physical function in older adults. Experimental Gerontology, 47(1), 38–44. 22015325 10.1016/j.exger.2011.10.001 PMC3245356

[eph13373-bib-0010] Carey, E. J. , Lai, J. C. , Wang, C. W. , Dasarathy, S. , Lobach, I. , Montano‐Loza, A. J. , & Dunn, M. A. (2017). A multicenter study to define sarcopenia in patients with end‐stage liver disease. Liver Transplantation, 23(5), 625–633. 28240805 10.1002/lt.24750 PMC5762612

[eph13373-bib-0011] Cruz‐Jentoft, A. J. , Bahat, G. , Bauer, J. , Boirie, Y. , Bruyère, O. , Cederholm, T. , Cooper, C. , Landi, F. , Rolland, Y. , Sayer, A. A. , Schneider, S. M. , Sieber, C. C. , Topinkova, E. , Vandewoude, M. , Visser, M. , & Zamboni, M. , Writing Group for the European Working Group on Sarcopenia in Older People 2 (EWGSOP2), and the Extended Group for EWGSOP2 . (2019). Sarcopenia: Revised European consensus on definition and diagnosis. Age and Ageing, 48(1), 16–31. 30312372 10.1093/ageing/afy169 PMC6322506

[eph13373-bib-0012] Dasarathy, S. , & Merli, M. (2016). Sarcopenia from mechanism to diagnosis and treatment in liver disease. Journal of Hepatology, 65(6), 1232–1244. 27515775 10.1016/j.jhep.2016.07.040 PMC5116259

[eph13373-bib-0013] Dhaliwal, A. , Williams, F. R. , Quinlan, J. I. , Allen, S. L. , Greig, C. , Filer, A. , Raza, K. , Ghosh, S. , Lavery, G. G. , Newsome, P. N. , Choudhary, S. , Breen, L. , Armstrong, M. J. , Elsharkawy, A. M. , & Lord, J. M. (2021). Evaluation of the mechanisms of sarcopenia in chronic inflammatory disease: Protocol for a prospective cohort study. Skeletal Muscle, 11(1), 1–14. 34895316 10.1186/s13395-021-00282-5 PMC8665319

[eph13373-bib-0014] Franchi, M. V. , Longo, S. , Mallinson, J. , Quinlan, J. I. , Taylor, T. , Greenhaff, P. L. , & Narici, M. V. (2018). Muscle thickness correlates to muscle cross‐sectional area in the assessment of strength training‐induced hypertrophy. Scandinavian Journal of Medicine & Science in Sports, 28(3), 846–853. 28805932 10.1111/sms.12961 PMC5873262

[eph13373-bib-0015] Franchi, M. V. , Reeves, N. D. , & Narici, M. V. (2017). Skeletal muscle remodeling in response to eccentric vs. concentric loading: Morphological, molecular, and metabolic adaptations. Frontiers in Physiology, 8, 1–16. 28725197 10.3389/fphys.2017.00447 PMC5495834

[eph13373-bib-0016] Giusto, M. , Lattanzi, B. , Albanese, C. , Galtieri, A. , Farcomeni, A. , Giannelli, V. , Lucidi, C. , Di Martino, M. , Catalano, C. , & Merli, M. (2015). Sarcopenia in liver cirrhosis: The role of computed tomorography scan for the assessment of muscle mass compared with dual‐energy X‐ray absorptiometry and anthriopometry. European Journal of Gastroenterology & Hepatology, 27(3), 328–334. 25569567 10.1097/MEG.0000000000000274

[eph13373-bib-0017] Hardy, E. J. O. , Inns, T. B. , Hatt, J. , Doleman, B. , Bass, J. J. , Atherton, P. J. , Lund, J. N. , & Phillips, B. E. (2022). The time course of disuse muscle atrophy of the lower limb in health and disease. Journal of Cachexia, Sarcopenia and Muscle, 13(6), 2616–2629. 36104842 10.1002/jcsm.13067 PMC9745468

[eph13373-bib-0018] Janssen, I. , Heymsfield, S. B. , Wang, Z. , & Ross, R. (2000). Skeletal muscle mass and distribution in 468 men and women aged 18–88 yr. Journal of Applied Physiology, 89(1), 81–88. 10904038 10.1152/jappl.2000.89.1.81

[eph13373-bib-0019] Kim, G. , Kang, S. H. , Kim, M. Y. , & Baik, S. K. (2017). Prognostic value of sarcopenia in patients with liver cirrhosis: A systematic review and meta‐analysis. PLoS ONE, 12(10), 1–16. 10.1371/journal.pone.0186990 PMC5655454 29065187

[eph13373-bib-0020] Lai, J. C. , Covinsky, K. E. , Dodge, J. L. , Boscardin, W. J. , Segev, D. L. , Roberts, J. P. , & Feng, S. (2017). Development of a novel frailty index to predict mortality in patients with end‐stage liver disease. Hepatology, 66(2), 564–574. 28422306 10.1002/hep.29219 PMC5519430

[eph13373-bib-0021] Lai, J. C. , Tandon, P. , Bernal, W. , Tapper, E. B. , Ekong, U. , Dasarathy, S. , & Carey, E. J. (2021). Malnutrition, frailty, and sarcopenia in patients with cirrhosis: 2021 Practice guidance by the American Association for the study of liver diseases. Hepatology, 74(3), 1611–1644. 34233031 10.1002/hep.32049 PMC9134787

[eph13373-bib-0022] Larsson, L. , Degens, H. , Li, M. , Salviati, L. , Lee, Y. I. , Thompson, W. , Kirkland, J. L. , & Sandri, M. (2019). Sarcopenia: Aging‐related loss of muscle mass and function. Physiological Reviews, 99(1), 427–511. 30427277 10.1152/physrev.00061.2017 PMC6442923

[eph13373-bib-0023] Linge, J. , Ekstedt, M. , & Dahlqvist Leinhard, O. (2021). Adverse muscle composition is linked to poor functional performance and metabolic comorbidities in NAFLD. JHEP Reports, 3(1), 100197. 33598647 10.1016/j.jhepr.2020.100197 PMC7868647

[eph13373-bib-0024] Maden‐Wilkinson, T. M. , Balshaw, T. G. , Massey, G. J. , & Folland, J. P. (2020). What makes long‐term resistance‐trained individuals so strong? A comparison of skeletal muscle morphology, architecture, and joint mechanics. Journal of Applied Physiology, 128(4), 1000–1011. 31873069 10.1152/japplphysiol.00224.2019 PMC7191505

[eph13373-bib-0025] McGlory, C. , Gorissen, S. H. M. , Kamal, M. , Bahniwal, R. , Hector, A. J. , Baker, S. K. , Chabowski, A. , & Phillips, S. M. (2019). Omega‐3 fatty acid supplementation attenuates skeletal muscle disuse atrophy during two weeks of unilateral leg immobilization in healthy young women. FASEB Journal, 33(3), 4586–4597. 30629458 10.1096/fj.201801857RRR

[eph13373-bib-0026] McGregor, R. A. , Cameron‐Smith, D. , & Poppitt, S. D. (2014). It is not just muscle mass: A review of muscle quality, composition and metabolism during ageing as determinants of muscle function and mobility in later life. Longevity & Healthspan, 3(1), 9. 25520782 10.1186/2046-2395-3-9 PMC4268803

[eph13373-bib-0027] Mitchell, W. K. , Williams, J. , Atherton, P. , Larvin, M. , Lund, J. , & Narici, M. (2012). Sarcopenia, dynapenia, and the impact of advancing age on human skeletal muscle size and strength; a quantitative review. Frontiers in Physiology, 3, 260. 22934016 10.3389/fphys.2012.00260 PMC3429036

[eph13373-bib-0028] Montano‐Loza, A. J. , Angulo, P. , Meza‐Junco, J. , Prado, C. M. M. , Sawyer, M. B. , Beaumont, C. , Esfandiari, N. , Ma, M. , & Baracos, V. E. (2016). Sarcopenic obesity and myosteatosis are associated with higher mortality in patients with cirrhosis. Journal of Cachexia, Sarcopenia and Muscle, 7(2), 126–135. 27493866 10.1002/jcsm.12039 PMC4864157

[eph13373-bib-0029] Monti, E. , Franchi, M. V. , Badiali, F. , Quinlan, J. I. , Longo, S. , & Narici, M. V. (2020). The time‐course of changes in muscle mass, architecture and power during 6 weeks of plyometric training. Frontiers in Physiology, 11, 1–14. 32848873 10.3389/fphys.2020.00946 PMC7417646

[eph13373-bib-0030] Narici, M. , Franchi, M. , & Maganaris, C. (2016). Muscle structural assembly and functional consequences. Journal of Experimental Biology, 219(2), 276–284. 26792340 10.1242/jeb.128017

[eph13373-bib-0031] Ogawa, M. , Yoshiko, A. , Tanaka, N. , Koike, T. , Oshida, Y. , & Akima, H. (2020). Comparing intramuscular adipose tissue on T1‐weighted and two‐point Dixon images. PLoS ONE, 15(4), 1–15. 10.1371/journal.pone.0231156 PMC7144956 32271803

[eph13373-bib-0032] Pasco, J. A. , Stuart, A. L. , Holloway‐Kew, K. L. , Tembo, M. C. , Sui, S. X. , Anderson, K. B. , Hyde, N. K. , Williams, L. J. , & Kotowicz, M. A. (2020). Lower‐limb muscle strength: Normative data from an observational population‐based study. BMC Musculoskeletal Disorders, 21(1), 4–11. 32035479 10.1186/s12891-020-3098-7 PMC7007641

[eph13373-bib-0033] Quinlan, J. I. , Franchi, M. V. , Gharahdaghi, N. , Badiali, F. , Francis, S. , Hale, A. , Phillips, B. E. , Szewczyk, N. , Greenhaff, P. L. , Smith, K. , Maganaris, C. , Atherton, P. J. , & Narici, M. V. (2021). Muscle and tendon adaptations to moderate load eccentric vs. concentric resistance exercise in young and older males. GeroScience, 43(4), 1567–1584. 34196903 10.1007/s11357-021-00396-0 PMC8492846

[eph13373-bib-0034] Quinlan, J. I. , Jones, C. , Bissonnette, E. , Dhaliwal, A. , Williams, F. , Choudhary, S. , Breen, L. , Lavery, G. G. , Armstrong, M. J. , Elsharkawy, A. M. , Lord, J. M. , & Greig, C. A. (2022). The impact of slice interval and equation on the accuracy of magnetic resonance image estimation of quadriceps muscle volume in end stage liver disease. Frontiers in Rehabilitation Sciences, 3, 1–9. 10.3389/fresc.2022.854041 PMC9397895 36189070

[eph13373-bib-0035] Reid, K. , & Fielding, R. (2012). Skeletal muscle power: A critical determinant of physical functioning in older adults. Exercise and Sport Sciences Reviews, 40(1), 4–12. 22016147 10.1097/JES.0b013e31823b5f13 PMC3245773

[eph13373-bib-0036] Rowlands, A. V. , Yates, T. , Davies, M. , Khunti, K. , & Edwardson, C. L. (2016). Raw accelerometer data analysis with GGIR R‐package: Does accelerometer brand matter? Medicine and Science in Sports and Exercise, 48(10), 1935–1941. 27183118 10.1249/MSS.0000000000000978

[eph13373-bib-0037] Sarto, F. , Spörri, J. , Fitze, D. P. , Quinlan, J. I. , Narici, M. V. , & Franchi, M. V. (2021). Implementing ultrasound imaging for the assessment of muscle and tendon properties in elite sports: Practical aspects, methodological considerations and future directions. Sports Medicine, 51(6), 1151–1170. 33683628 10.1007/s40279-021-01436-7 PMC8124062

[eph13373-bib-0038] Schaap, L. A. , Van Schoor, N. M. , Lips, P. , & Visser, M. (2018). Associations of sarcopenia definitions, and their components, with the incidence of recurrent falling and fractures: The longitudinal aging study Amsterdam. Journals of Gerontology – Series A Biological Sciences and Medical Sciences, 73(9), 1199–1204. 29300839 10.1093/gerona/glx245

[eph13373-bib-0039] Seynnes, O. R. , & Cronin, N. J. (2020). Simple Muscle Architecture Analysis (SMA): An ImageJ macro tool to automate measurements in B‐mode ultrasound scans. PLoS ONE, 15(2), e0229034. 32049973 10.1371/journal.pone.0229034 PMC7015391

[eph13373-bib-0040] Tandon, P. , Low, G. , Mourtzakis, M. , Zenith, L. , Myers, R. P. , Abraldes, J. G. , Shaheen, A. A. M. , Qamar, H. , Mansoor, N. , Carbonneau, M. , Ismond, K. , Mann, S. , Alaboudy, A. , & Ma, M. (2016). A model to identify sarcopenia in patients with cirrhosis. Clinical Gastroenterology and Hepatology, 14(10), 1473–1480.e3. 27189915 10.1016/j.cgh.2016.04.040

[eph13373-bib-0041] Tuttle, L. J. , Sinacore, D. R. , & Mueller, M. J. (2012). Intermuscular adipose tissue is muscle specific and associated with poor functional performance. Journal of Aging Research, 2012, 1–7. 10.1155/2012/172957 PMC3361226 22666591

[eph13373-bib-0042] Volpi, E. , Nazemi, R. , & Fujita, S. (2004). Muscle tissue changes with aging. Current Opinion in Clinical Nutrition and Metabolic Care, 7(4), 405–410. 15192443 10.1097/01.mco.0000134362.76653.b2 PMC2804956

[eph13373-bib-0043] Wilhelm, E. N. , Rech, A. , Minozzo, F. , Radaelli, R. , Botton, C. E. , & Pinto, R. S. (2014). Relationship between quadriceps femoris echo intensity, muscle power, and functional capacity of older men. Age (Omaha), 36(3), 1113–1122. 10.1007/s11357-014-9625-4 PMC4082605 24515898

[eph13373-bib-0044] Williams, F. R. , Milliken, D. , Lai, J. C. , & Armstrong, M. J. (2021). Assessment of the frail patient with end‐stage liver disease: A practical overview of sarcopenia, physical function, and disability. Hepatology Communications, 5(6), 923–937. 34141980 10.1002/hep4.1688 PMC8183168

[eph13373-bib-0045] Zamboni, M. , Gattazzo, S. , & Rossi, A. P. (2019). Myosteatosis: A relevant, yet poorly explored element of sarcopenia. European Geriatric Medicine, 10(1), 5–6. 32720282 10.1007/s41999-018-0134-3

